# Enhancing Biodegradability of Poly(L-Lactic Acid) via Incorporation of Thermoplastic Chitosan

**DOI:** 10.3390/polym18111404

**Published:** 2026-06-05

**Authors:** Yodthong Baimark, Prasong Srihanam, Yaowalak Srisuwan

**Affiliations:** Biodegradable Polymers Research Unit, Department of Chemistry and Centre of Excellence for Innovation in Chemistry, Faculty of Science, Mahasarakham University, Maha Sarakham 44150, Thailand; prasong.s@msu.ac.th (P.S.); yaowalak.s@msu.ac.th (Y.S.)

**Keywords:** poly(lactic acid), chitosan, biocomposite, mechanical properties, biodegradation

## Abstract

Poly(L-lactic acid) (PLLA) is a biodegradable polyester that has garnered widespread attention for its potential applications as a replacement for conventional petroleum-based plastics. However, PLLA’s prolonged biodegradation is a significant limitation in its applications, particularly in single-use packaging, as it can lead to environmental accumulation and hinder the sustainability goals of reducing plastic waste. This paper examines the effect of incorporating thermoplastic chitosan (TPC) on the mechanical and biodegradation properties of PLLA. TPC was prepared using lactic acid as a plasticizer. PLLA/TPC composites were produced by thermo-mechanical processes. TPC contents of 1%, 2.5%, 5%, and 10% were investigated. The PLLA/TPC films exhibited distinct phase separation, as verified by scanning electron microscopy analysis. The incorporation of 2.5% TPC led to a 20.8% enhancement in elongation at break and a 7.4% improvement in tensile toughness relative to pure PLLA film. Nonetheless, both values diminished when the TPC content surpassed 2.5 wt%. The surface wettability of the PLLA/TPC films, assessed via water contact angle measurements and weight loss from soil burial tests, enhanced with greater TPC content. The PLLA/TPC films showed significantly greater weight loss after being buried in soil for 12 months compared to pure PLLA film. The increases in weight loss were 4, 11, 14, and 72 times greater for the TPC contents of 1%, 2.5%, 5%, and 10%, respectively. Incorporating TPC in this study improved the flexibility and biodegradability of PLLA, leading to PLLA-based composites with enhanced potential for environmentally sustainable single-use packaging.

## 1. Introduction

Interest in biodegradable bioplastics is steadily increasing as alternatives to conventional petroleum-based plastics, aiming to mitigate plastic waste pollution and lower carbon footprints [1,2,3]. Poly(L-lactic acid) (PLLA) is a bio-based polymer derived from renewable resources like corn starch (Ingeo™ PLLA) and sugarcane (Luminy™ PLLA). Due to its excellent biocompatibility, compostability, high strength, and commercial availability [4,5,6], PLLA is widely utilized in tissue engineering, drug delivery, automotive components, and packaging [7,8,9,10]. However, a major drawback of PLLA is its slow biodegradation rate, particularly in landfills, which stems from its highly hydrophobic molecular structure [11,12]. This limitation restricts its viability for single-use packaging applications.

Numerous studies have aimed to accelerate the biodegradation rate and reduce the production costs of PLLA by blending it with abundant hydrophilic biopolymers, such as thermoplastic starch [13,14,15], thermoplastic alginate [16], and chitosan [17,18,19]. These approaches have the potential to yield environmentally sustainable PLLA-based composites that serve as promising, versatile alternatives to petroleum-based plastics in various applications, including packaging, agricultural films, and disposable cutlery. Consequently, research focusing on developing novel PLLA-based composites through diverse biopolymer blending remains essential for expanding the scope of alternative bioplastics.

Chitosan, a renewable and abundant biopolymer derived from the deacetylation of chitin, is a primary component of shrimp and crab shells [20,21]. Due to its biodegradability, non-toxicity, biocompatibility, and antimicrobial properties [22,23,24,25,26,27], chitosan has been extensively investigated for applications in wound dressings, drug delivery, and food packaging. To facilitate straightforward processing via conventional techniques like thermo-compression, several studies have reported the preparation of thermoplastic chitosan (TPC) using appropriate plasticizers such as lactic acid [28,29,30,31]. As a hydrophilic biopolymer, TPC is expected to enhance the biodegradation rate of PLLA when blended.

For instance, Kamaludin et al. [18] prepared PLLA/chitosan powder biocomposites and demonstrated that incorporating 2.5 parts per hundred of resin (phr) increased the tensile strength and tensile modulus by merely 2% and 14%, respectively, compared to neat PLLA, alongside increasing water affinity. However, such particulate biocomposites often exhibit limited elasticity enhancement due to interfacial phase separation. To address these limitations, the utilization of TPC as a functional blending component offers a distinct pathway to tailor both ductile properties and environmental degradation pathways. To date, no systematic studies have reported on the preparation and physicochemical characterization of PLLA/TPC composite systems, marking the distinct novelty of this work. Therefore, this research investigates PLLA/TPC films prepared via melt-blending, where TPC was plasticized with lactic acid. The influence of TPC content on the thermal, phase morphological, mechanical, surface wettability, and biodegradation properties of the resulting films was systematically evaluated.

## 2. Materials and Methods

### 2.1. Materials

Poly(L-lactic acid) (PLLA) grade 3251D was purchased from NatureWorks LLC (Waltham, MA, USA). The melt flow rate is approximately 30 g/10 min at 190 °C under a load of 2.16 kg. Chitosan powder was purchased from Sinudom Agriculture Ltd. Part. (Suratthani, Thailand). It has a viscosity of 300 cps (measured at 20 °C from a 2 wt% chitosan solution in a 1 wt% acetic acid aqueous solution) and a 94% degree of deacetylation. An 88 wt% L-lactic acid (LLA) solution was obtained from Purac in Rayong, Thailand.

### 2.2. Preparation of PLLA/TPC Films

Thermoplastic chitosan (TPC) was synthesized by kneading and rolling chitosan powder with L-lactic acid (LLA) at a 50/50 *w*/*w* ratio, following our previously reported procedure [31]. Prior to blending, neat PLLA and TPC were dried in an airflow oven at 80 °C for 6 h. Melt-blending was performed using a Polylab OS System internal mixer (HAAKE, Waltham, MA, USA) at 170 °C and a rotor speed of 100 rpm for 10 min. Blends with PLLA/TPC weight ratios of 100/0, 99/1, 97.5/2.5, 95/5, and 90/10 *w*/*w* were prepared. The resulting PLLA/TPC pellets were dried again at 80 °C for 6 h and then fabricated into films via compression molding using an Auto CH compression molding machine (Carver, Wabash, IN, USA). The pellets were preheated at 175 °C for 3 min, compressed under a force of 5 MPa for another 3 min, and subsequently cooled between water-cooled plates under the same pressure for 3 min. The thickness of the fabricated films ranged between 0.2 and 0.3 mm. All film samples were stored in a desiccator for 24 h prior to characterization.

### 2.3. Characterization of PLLA/TPC Films

#### 2.3.1. Fourier Transform Infrared Analysis

The chemical functional groups in the film samples were identified using an INVENIO-S Fourier transform infrared (FTIR) spectrometer (Bruker Corporation, Karlsruhe, Germany) that was equipped with an attenuated total reflectance (ATR) mode. The FTIR spectra were obtained over a frequency range from 500 cm^−1^ to 4000 cm^−1^, with a resolution of 4 cm^−1^ and an accumulation of 32 scans.

#### 2.3.2. Differential Scanning Calorimetry

The thermal transition properties of the film samples were analyzed using a Pyris Diamond differential scanning calorimeter (PerkinElmer, Waltham, MA, USA) in a nitrogen atmosphere. Prior to testing, the thermal history of the films was eliminated by heating them at 200 °C for three minutes. Then cool to 0 °C at a rate of 100 °C/min. Following this, the samples were scanned from 0 °C to 200 °C at a heating rate of 10 °C/min. The degree of crystallinity (Xc) of the PLLA was determined using Equation (1), incorporating the enthalpies of melting (ΔHm) and cold crystallization (ΔHcc) values.

X_c_ (%) = [(ΔH_m_ − ΔH_cc_)/(93.6 × W_PLA_)] × 100 (1)

where 93.6 J/g refers to the ΔH_m_ associated with 100% X_c_ of PLLA [16]. W_PLA_ denotes the weight fraction of PLLA.

#### 2.3.3. Thermogravimetric Analysis

The thermal stability of film samples was measured by an SDT Q600 thermogravimetric analyzer (TA-Instruments, New Castle, DE, USA) from 50 to 800 °C under a nitrogen flow of 100 mL/min. A heating rate was 20 °C/min.

#### 2.3.4. Scanning Electron Microscopy

The microstructures of fractured surfaces of film samples were analyzing using a TM4000Plus scanning electron microscope (HITACHI, Tokyo, Japan) at an acceleration voltage of 15 kV. The film samples were broken after being immersed in liquid nitrogen for 10 min. Prior to imaging, the film samples underwent sputter-coated with gold.

#### 2.3.5. Tensile Test

Tensile properties of the film samples were measured by a LY-1066B universal testing machine (Dongguan Liyi Environmental Technology Co., Ltd., Dongguan, China) at 25 °C and 50% RH in accordance with the American Society for Testing and Materials (ASTM) standard test method D882 [32]. The specimen size for the tensile test was 10 mm in width and 100 mm in length. The initial grip separation was set at 50 mm, and the specimens were stretched at a crosshead speed of 50 mm/min. Tensile toughness was determined from the area under the stress–strain curve. Five repetitions were carried out for each film sample formulation.

#### 2.3.6. Water Contact Angle Test

The water contact angle of the film samples was measured by the sessile drop method using an OCA11 contact angle analyzer (DataPhysics Instruments, Filderstadt, Germany). For this method, 2.5 µL of deionized water was dropped on the film surfaces. The water contact angles from the left and right sides of the water droplet were measured after 15 s and then averaged. The measurement was taken in triplicate at 25 °C.

#### 2.3.7. Biodegradation Test

Biodegradation of film samples was investigated using a burial test based on established methods [16]. Film samples, measuring 15 mm × 15 mm, were dried at 105 °C for 24 h prior to weighing (W_i_). These film samples were then placed in nylon mesh bags with a mesh size of 1.0 mm and buried 5 cm beneath the soil surface. The soil used in the experiment was general-purpose potting soil. Watering occurred every other day over a period of 12 months. Soil temperature varied between 25 °C and 30 °C, while the pH ranged from 6.0 to 7.0. Soil moisture levels were maintained between 50% and 60%. Every two months, the film samples were retrieved, washed with water, dried again at 105 °C for 24 h, and weighed (W_f_). The weight loss of the film samples was calculated using Equation (2). Three replicates were conducted for each film sample to obtain an average result.

Weight loss (%) = [(W_i_ × W_f_)/W_i_] × 100 (2)

## 3. Results and Discussion

### 3.1. FTIR Analysis

**Figure 1:**
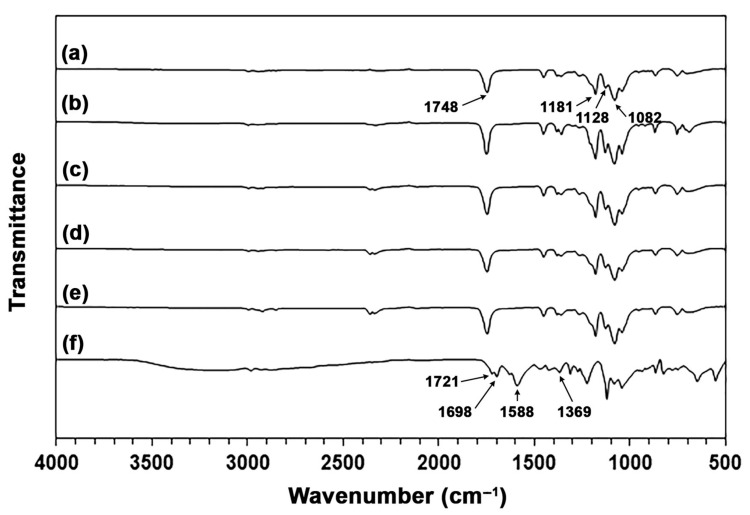
Typical ATR-FTIR spectra of (a) pure PLLA film and PLLA/TPC films with PLLA/TPC ratios of (b) 99/1, (c) 97.5/2.5, (d) 95/5, and (e) 90/10% *w*/*w*, and (f) TPC.

ATR-FTIR spectroscopy was employed to analyze the chemical structures of the polymer composites, with the representative spectra of neat PLLA, TPC, and PLLA/TPC films shown in Figure 1. The neat PLLA film (Figure 1(a)) exhibited a characteristic carbonyl (C=O) stretching band at 1748 cm^−1^, ester C–O–C stretching bands at 1181, 1128, and 1082 cm^−1^, and C–H stretching bands at 2998, 2944, and 2878 cm^−1^ [33,34]. The TPC spectrum (Figure 1(f)) displayed broad, overlapping O–H and N–H stretching bands in the 3000–3600 cm^−1^ region [35], C–H stretching bands between 2800 and 3000 cm^−1^ [36], a carbonyl band corresponding to lactic acid at 1721 cm^−1^ [30], and amide I (C=O stretching), amide II (N–H bending), and amide III (C–N stretching) bands at 1698, 1588, and 1369 cm^−1^, respectively [37,38,39].

The ATR-FTIR spectra of all PLLA/TPC films closely resembled that of the neat PLLA film, showing no detectable shifts in the PLLA absorption bands or distinct characteristic bands of TPC. This behavior is likely attributed to the low TPC content, which was insufficient to resolve the absorption bands associated with chitosan and lactic acid. For instance, the PLLA/10%TPC film contains only 5 wt% each of chitosan and lactic acid.

### 3.2. Differential Scanning Calorimetry (DSC)

**Figure 2:**
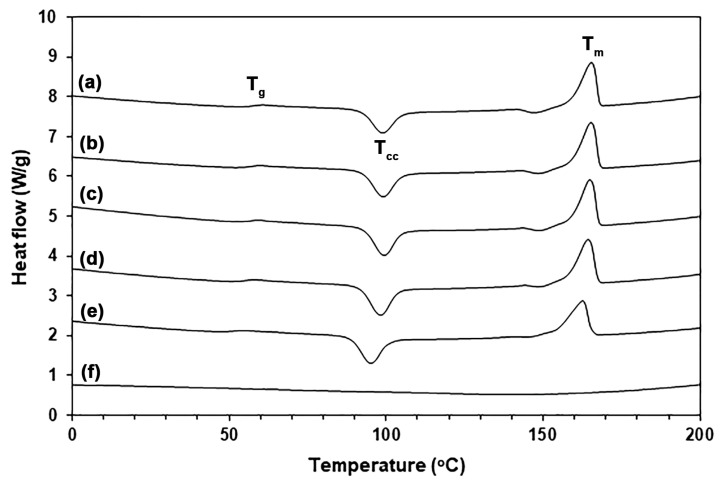
DSC thermograms of (a) pure PLLA film and PLLA/TPC films with PLLA/TPC ratios of (b) 99/1, (c) 97.5/2.5, (d) 95/5, and (e) 90/10% *w*/*w*, and (f) TPC.

DSC thermograms were obtained to investigate the effect of TPC addition on the thermal transition properties of the PLLA matrix, as shown in Figure 2 and summarized in Table 1. Neat PLLA and all PLLA/TPC films exhibited a glass transition temperature (T_g_) step, an exothermic cold crystallization peak (T_cc_), and an endothermic melting peak (T_m_). The neat PLLA film displayed a T_g_ of 57 °C, a T_cc_ of 99 °C, and a T_m_ of 165 °C. In contrast, TPC showed no thermal transitions within the 0–200 °C range. As the TPC content increased, the T_g_ of the PLLA/TPC films progressively decreased. This reduction is likely attributed to the diffusion of L-lactic acid (LLA) plasticizer from the TPC phase into the PLLA matrix. LLA inserts itself between the polymer chains, increasing free volume and reducing intermolecular forces, which ultimately leads to a lower T_g_ value [40,41].

The T_cc_ of the PLLA/TPC films decreased upon incorporating 5% and 10% TPC. This reduction is attributed to the diffused LLA at these concentrations, which enhances the mobility and rearrangement of PLLA chains during cold crystallization upon heating, thereby lowering the T_cc_ [42]. Similarly, the T_m_ of the films declined with 5% and 10% TPC additions. This decrease likely stems from the infiltration of diffused LLA, which induces the formation of less perfect PLLA crystals with lower thermal stability [43]. The degree of crystallinity (X_c_) of the neat PLLA film, calculated using Equation (1), was 11.9% (Table 1). The addition of TPC above 1 wt% resulted in a slight decrease in the X_c_ of PLLA, with values shifting from 11.9% to approximately 7.2–7.5%. This minor reduction indicates that elevated TPC concentrations may hinder the mobility of PLLA chains during the crystallization process. However, further statistical validation is necessary to substantiate the presence of a significant plasticizing effect that restricts crystal growth.

### 3.3. Thermogravimetric Analysis (TGA)

**Figure 3:**
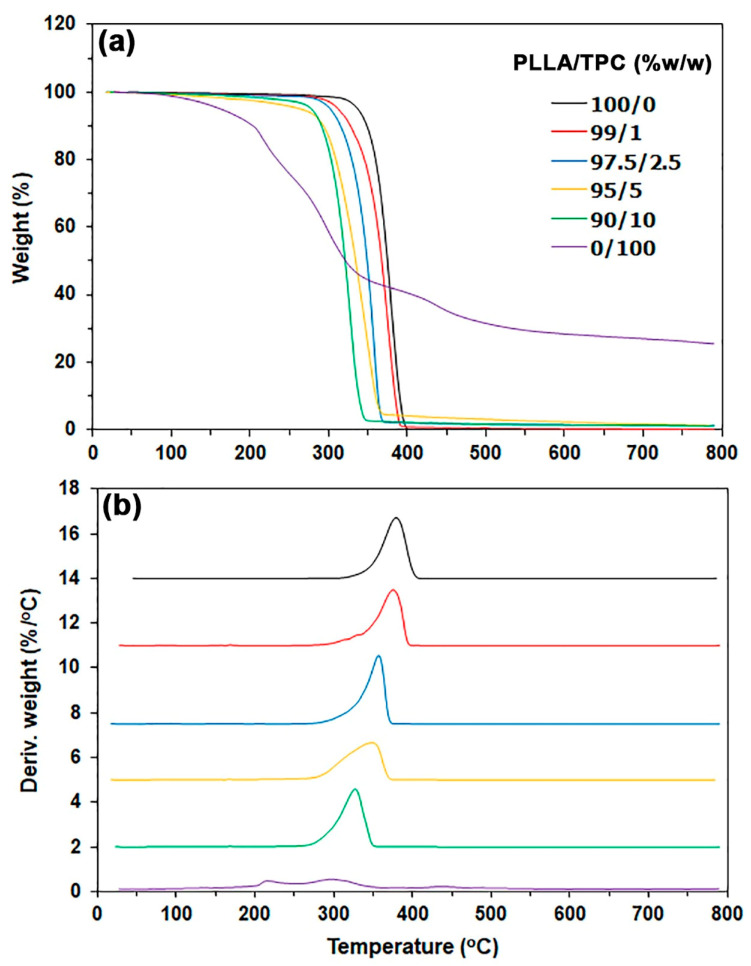
(**a**) TG and (**b**) DTG thermograms of PLLA film, PLLA/TPC films, and TPC.

Thermogravimetric (TG) and derivative thermogravimetric (DTG) analyses were conducted to evaluate the effect of TPC content on the thermal stability of the PLLA/TPC films, as shown in Figure 3a and Figure 3b, respectively. The corresponding TGA parameters are summarized in Table 2. The TG thermogram of the neat PLLA film (Figure 3a) reveals a single-step thermal degradation behavior within 320–420 °C, corresponding to the decomposition of the PLLA backbone, which agrees with previous reports [44,45]. In contrast, TPC exhibits a three-step thermal degradation profile: the first stage (50–150 °C) involves the evaporation of residual moisture; the second stage (150–250 °C) is attributed to the volatilization of LLA; and the third stage (250–400 °C) corresponds to the cleavage of glycosidic bonds in the chitosan backbone [46,47]. The PLLA/TPC films display a single-step degradation process, with a noticeable trend toward accelerated thermal degradation as the TPC content increases.

As presented in Table 2, the decomposition temperatures at 5% weight loss (T_5%_), 10% weight loss (T_10%_), and 50% weight loss (T_50%_), along with the maximum degradation rate temperature (T_max_), consistently shifted toward lower values with increasing TPC content. These findings align with Kamaludin et al. [18], who reported a reduction in the thermal stability of the PLLA matrix upon the incorporation of chitosan powder. In addition, the presence of diffused LLA within the PLLA matrix likely disrupts the intermolecular forces between the PLLA chains, thereby undermining thermal stability. This behavior is consistent with previous studies demonstrating that diffused glycerol from thermoplastic starch [48] and thermoplastic alginate [16] similarly reduces PLLA thermal stability by disrupting intermolecular interactions. Additionally, the char residue at 800 °C progressively increased with higher TPC content; TPC yielded a char residue of 25.4%, whereas neat PLLA exhibited none. Crucially, the thermal degradation temperatures of all PLLA/TPC films remained well above the compression molding temperature of 175 °C. This confirms that the incorporation of TPC does not compromise the thermal stability of the composites during melt processing.

### 3.4. Microstructure of Fractured Surfaces

**Figure 4:**
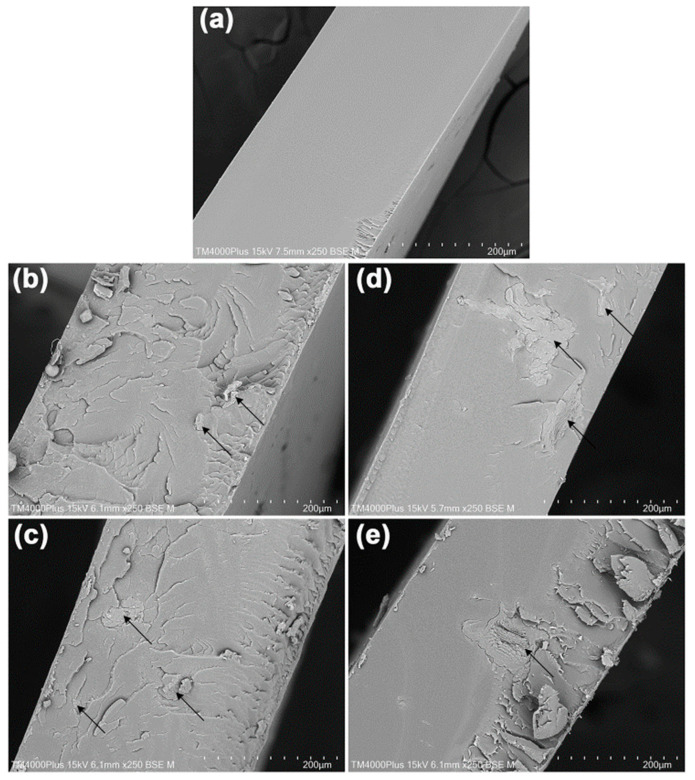
SEM images of cryofractured surfaces of (**a**) pure PLLA film and PLLA/TPC films with PLLA/TPC ratios of (**a**) 100/0, (**b**) 99/1, (**c**) 97.5/2.5, (**d**) 95/5, and (**e**) 90/10% *w*/*w*. All bar scales = 200 µm. Some TPC phases were indicated by black arrows.

The microstructures of the PLLA/TPC composites were analyzed using cryofractured film surfaces, as illustrated in Figure 4. The neat PLLA film (Figure 4a) displayed a smooth fractured surface, characteristic of its brittle nature. In contrast, the fractured surfaces of the PLLA/TPC films (Figure 4b–e) clearly revealed distinct dispersed TPC phases. The domain size of the TPC phases increased with higher TPC content, which is likely attributed to enhanced phase aggregation driven by the hydrophilic-hydrophobic mismatch between PLLA and TPC. Chitosan possesses a backbone rich in hydrophilic functional groups, specifically hydroxyl and amino groups. Furthermore, in the presence of lactic acid, these amino groups can protonate into highly hydrophilic, positively charged of –NH_3_^+^ groups. Conversely, PLLA contains numerous hydrophobic methyl side chains. These findings align with previous reports on PLLA composites incorporating hydrophilic biopolymers, such as thermoplastic starch [49] and thermoplastic alginate [16].

Visible gaps at the interfaces between the TPC and PLLA phases signify poor interfacial compatibility arising from their disparate hydrophilicity. The fractured surfaces of the PLLA/1%TPC and PLLA/2.5%TPC films exhibited greater roughness than the neat PLLA film. This increased roughness typically indicates enhanced energy dissipation during fracturing, which often correlates with improved material toughness. Conversely, the fractured surfaces of the PLLA/TPC films with TPC contents exceeding 2.5% became smoother, suggesting a reduction in toughness and elasticity. The correlations between these morphological changes and the mechanical performance of the PLLA/TPC films are evaluated in detail via tensile testing in the subsequent section.

### 3.5. Tensile Properties

**Figure 5:**
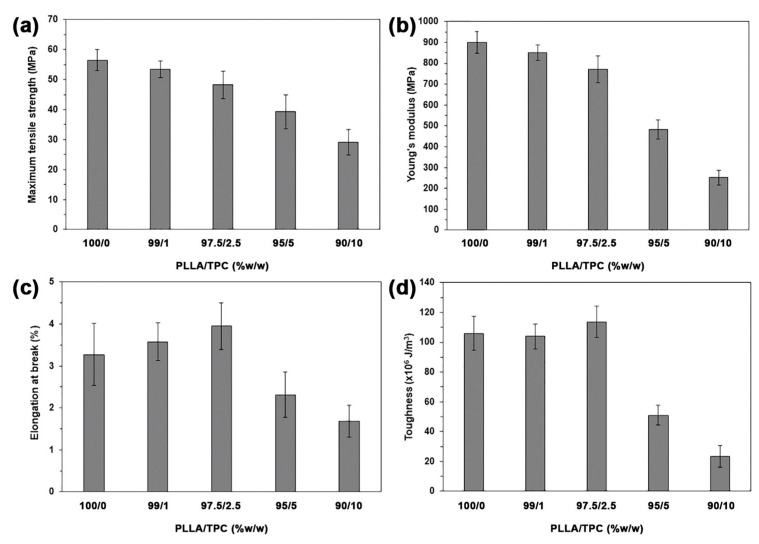
Tensile properties of pure PLLA and PLLA/TPC films: (**a**) maximum tensile strength, (**b**) Young’s modulus, (**c**) elongation at break, and (**d**) tensile toughness.

The mechanical performance of the PLLA/TPC films, including tensile strength, Young’s modulus, elongation at break, and tensile toughness, is presented in Figure 5. The neat PLLA film exhibited a tensile strength of 56.5 MPa, a Young’s modulus of 898.6 MPa, an elongation at break of 3.3%, and a tensile toughness of 105.9 × 10^6^ J/m^3^. As shown in Figure 5a,b, both tensile strength and Young’s modulus continuously decreased with increasing TPC content. This downward trend is attributed to the dispersed TPC phases acting as stress concentration sites, which significantly reduced the strength and stiffness of the composites. In our previous study, compression-molded TPC films demonstrated a lower tensile strength (11.5 MPa) and Young’s modulus (110.8 MPa) than neat PLLA. Furthermore, severe phase aggregation within polymer composites is known to create defect centers that accelerate mechanical failure [50]. This aligns with the SEM observations, where the dispersed TPC phases aggregated into larger domains at higher concentrations. Additionally, the migration of LLA from the TPC phase into the PLLA matrix induced a plasticization effect, further decreasing both the tensile strength and stiffness of the films.

The neat PLLA film exhibited an elongation at break of 3.3% and a tensile toughness of 105.9 J/m^3^. While these properties remained virtually unchanged upon incorporating 1% TPC, noticeable improvements were observed with the addition of 2.5% TPC (Figure 5c). Specifically, the PLLA/2.5%TPC film achieved an elongation at break of 4.0% and a tensile toughness of 113.7 J/m^3^, signifying enhanced flexibility and toughness relative to neat PLLA. This increased ductility is likely driven by the migration of LLA from the TPC phase, which exerted a plasticizing effect. The enhanced elongation at break and tensile toughness of the PLLA/2.5%TPC film is proposed to be associated with the localized plasticization induced by LLA migration. While direct tracking of LLA diffusion was not performed, the distinct decrease in T_g_ values combined with the increased matrix ductile tearing observed in the SEM images strongly indicates that LLA domains likely diffused from the TPC phase into the PLLA matrix, thereby improving chain mobility and mechanical performance. These results align with previous reports demonstrating that oligo-lactic acid incorporation effectively enhances PLLA film flexibility [51]. In contrast, the literature on PLLA/chitosan powder biocomposites indicates that introducing chitosan powder typically causes a monotonic decrease in elongation at break with increasing filler content [18]. This stark contrast further supports the premise that the diffused lactic acid within the TPC successfully plasticized and toughened the PLLA matrix.

However, both elongation at break and tensile toughness sharply declined when the TPC content exceeded 2.5 wt%. This deterioration implies that extensive aggregation of the TPC phase at higher loading levels created structural defects that accelerated fracture propagation under tensile stress. These mechanical trends are strongly corroborated by the SEM observations, which revealed that the cryofractured surfaces of the PLLA/5%TPC and PLLA/10%TPC films were significantly smoother than those of their lower-content counterparts (1% and 2.5% TPC).

### 3.6. Surface Wettability

The surface wettability of the PLLA/TPC films was evaluated via water contact angle measurements, as summarized in Table 3. A lower water contact angle signifies higher hydrophilicity and enhanced surface wettability. The neat PLLA film exhibited a water contact angle of 98.29°. In our previous study, compression-molded TPC films containing 50% LLA displayed a significantly lower contact angle of 60.1° [31], indicating that the TPC matrix possesses inherently higher surface wettability than neat PLLA. This characteristic is attributed to the molecular structure of chitosan, which is rich in hydrophilic functional groups, specifically amino and hydroxyl groups. These findings align with prior reports demonstrating that incorporating chitosan powder increases PLLA hydrophilicity [18], which is highly beneficial for accelerating subsequent environmental degradation. Furthermore, the presence of highly hydrophilic LLA within TPC contributes to this effect. Consequently, Table 3 illustrates that the water contact angle of the PLLA/TPC films progressively decreased, reflecting a corresponding increase in surface wettability, as the TPC content increased.

### 3.7. Biodegradability

**Figure 6:**
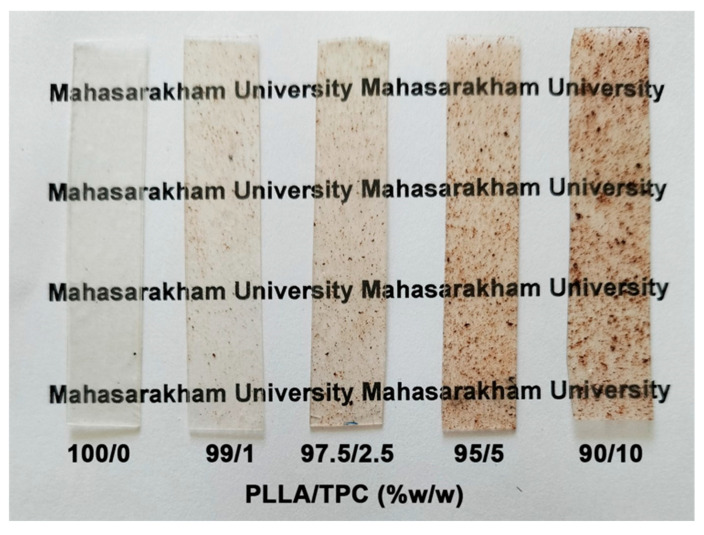
Visual appearances of pure PLLA and PLLA/TPC films.

The visual appearances of the neat PLLA and PLLA/TPC films are illustrated in Figure 6. The neat PLLA film appears clear and translucent. In contrast, the PLLA/TPC films exhibit distinct brown TPC domains dispersed throughout the PLLA matrix. As the TPC content increases, these TPC domains become noticeably larger. This phenomenon is attributed to the hydrophobic-hydrophilic mismatch between PLLA and TPC, as previously discussed in the surface wettability section, which induces more pronounced phase separation and larger domain sizes at higher TPC loading levels.

**Figure 7:**
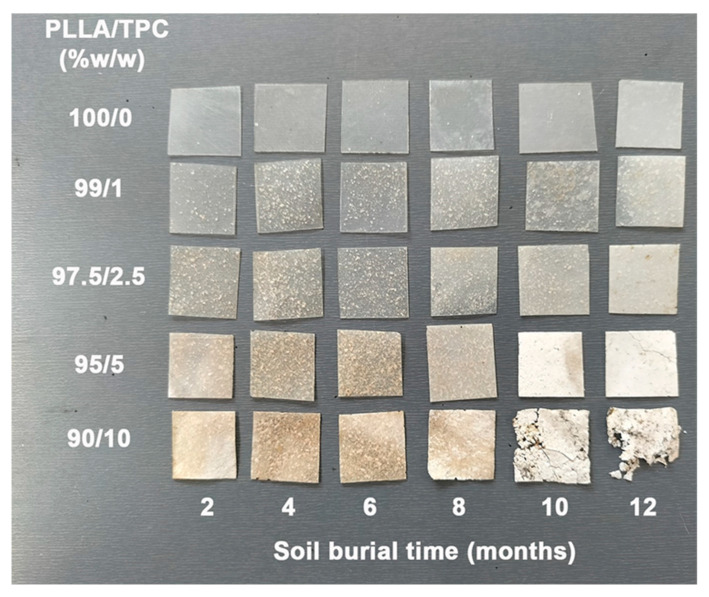
Visual appearances of pure PLLA and PLLA/TPC films after soil burial test.

The biodegradability of the film samples was assessed via a soil burial test, with the visual changes in the buried films over a 12-month period displayed in Figure 7. Both neat PLLA and PLLA/TPC films exhibited increased opacity with prolonged burial duration. This change is likely attributed to water absorption, which induced porosity and surface erosion [16,52]. The dispersed TPC domains transitioned from brown to opaque white after burial, signifying the preferential degradation of the TPC phase. The PLLA/5%TPC films fractured after 12 months of soil burial, whereas the PLLA/10%TPC films displayed significant disintegration and degradation after only 10 months.

**Figure 8:**
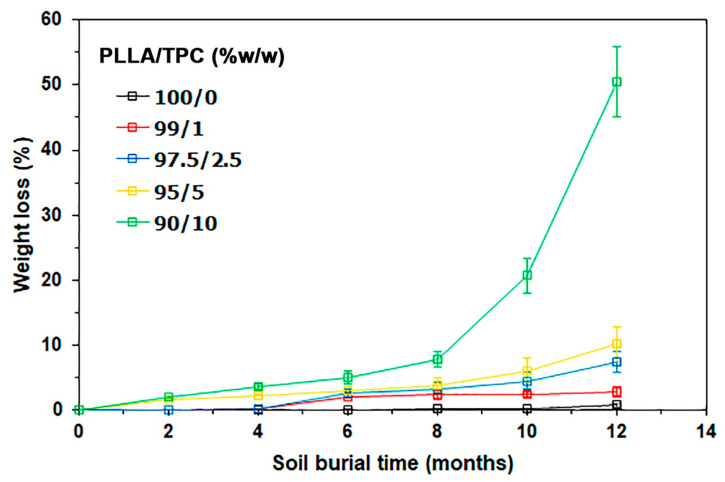
Weight losses of pure PLLA and PLLA/TPC films from soil burial test.

The biodegradability of the film samples was quantitatively analyzed via weight loss measurements at various time intervals, as shown in Figure 8. The neat PLLA film demonstrated the lowest weight loss after 12 months, reaching only 0.7%. This minimal weight loss is attributed to the highly hydrophobic nature of PLLA, which restricts the diffusion of water molecules into the polymer matrix [53]. Crucially, the weight loss of the composites progressively increased with both prolonged soil burial duration and higher TPC content.

After 12 months of soil burial, the PLLA/1%TPC, PLLA/2.5%TPC, PLLA/5%TPC, and PLLA/10%TPC films experienced weight losses of 2.8%, 7.4%, 10.1%, and 50.5%, respectively. These weight loss values exceeded the actual TPC content within each film, demonstrating that the PLLA matrix itself underwent degradation. These findings suggest that the incorporation of TPC effectively facilitates the biodegradation of PLLA. It is anticipated that the TPC domains within the composite films degrade preferentially. This initial erosion increases the surface area exposed to soil moisture, thereby accelerating the subsequent hydrolytic degradation of the PLLA matrix. After 12 months of soil burial, the degradation rates of PLLA/TPC films containing 1%, 2.5%, 5%, and 10% TPC were approximately 4, 11, 14, and 72 times faster, respectively, than the degradation rate of the neat PLLA film.

Both PLLA [54] and chitosan [55] are known to undergo biodegradation via a two-step mechanism. The initial stage involves abiotic hydrolysis triggered by soil moisture. Due to its superior hydrophilicity, TPC undergoes hydrolysis much faster than PLLA. The second stage consists of biotic degradation mediated by soil microorganisms. In the soil environment, PLLA is enzymatically cleaved by specific enzymes such as proteinase K (excreted by *Escherichia coli*), lipase (from *Bacillus* sp.), and esterases (produced by both *Bacillus* sp. and *Aspergillus westerdijkiae*) [56]. Concurrently, chitosan biodegradation is driven primarily by chitinase and chitosanase enzymes [57], which are produced by various soil microbes, including *Trichoderma* spp. fungi, as well as *Streptomyces* spp. and *Bacillus* spp. bacteria [57,58].

## 4. Conclusions

This research demonstrates a comprehensive approach to accelerating the biodegradation of poly(L-lactic acid) (PLLA)-based composites by incorporating thermoplastic chitosan (TPC) plasticized with L-lactic acid (LLA). DSC analysis revealed that TPC addition lowered the glass transition temperature (T_g_) of the PLLA matrix from 57 °C to 50 °C and reduced the degree of crystallinity (X_c_) from 11.9% to 7.2%, indicating effective plasticization. Although TGA showed a decrease in thermal stability with higher TPC loading, all composites remained thermally stable well above the processing temperature of 175 °C. Morphological analysis via SEM indicated poor interfacial compatibility between the phases, characterized by phase separation. Notably, cryofractured surfaces of the films containing 1% and 2.5% TPC exhibited enhanced roughness compared to neat PLLA and films with higher TPC contents. Tensile testing confirmed that incorporating 2.5% TPC optimized the ductility and toughness of the matrix, with elongation at break and tensile toughness increasing to 4.0% and 113.7 J/m^3^, respectively. Beyond 2.5 wt% TPC, these mechanical properties deteriorated due to extensive domain aggregation that formed structural defects. Water contact angle measurements demonstrated a progressive increase in surface wettability with higher TPC content, which subsequently accelerated the biodegradation rate during soil burial tests. Overall, the composite with 2.5% TPC emerged as the optimal formulation, yielding a 20.8% increase in elongation at break, a 7.4% increase in tensile toughness, and an 11-fold increase in biodegradation weight loss compared to neat PLLA. Although PLLA composites with higher TPC contents (5 wt% and 10 wt%) exhibit compromised mechanical properties, they demonstrate substantially accelerated biodegradation rates compared to both neat PLLA and the 2.5 wt% TPC formulation. Consequently, these high-TPC composites remain highly viable for specific single-use packaging sectors—such as short-lifecycle containers—where rapid environmental decomposition is prioritized over structural load-bearing capacity. Consequently, these PLLA/TPC composites offer significant potential as sustainable alternative materials for single-use packaging applications.

## Figures and Tables

**Table 1:** DSC data of pure PLLA and PLLA/TPC films.

PLLA/TPC (%*w*/*w*)	T_g_ (°C)	T_cc_ (°C)	ΔH_cc_ (J/g)	T_m_ (°C)	ΔH_m_ (J/g)	X_c_ (%)
100/0	57	99	28.1	165	39.2	11.9
99/1	55	99	32.0	165	40.6	9.3
97.5/2.5	54	99	33.7	165	40.3	7.2
95/5	53	98	34.1	164	41.1	7.5
90/10	50	95	32.0	162	38.2	7.4

**Table 2:** TGA data of pure PLLA film, PLLA/TPC films, and TPC.

PLLA/TPC (%*w*/*w*)	T_5%_ (°C) ^1^	T_10%_ (°C) ^1^	T_50%_ (°C) ^1^	Char Residue at 800 °C (%) ^1^	T_max_ (°C) ^2^
100/0	337	348	375	-	378
99/1	312	328	367	0.2	375
97.5/2.5	301	315	349	0.8	357
95/5	268	293	334	1.3	349
90/10	268	290	321	1.8	327
0/100	162	204	322	25.4	216, 297

^1^ obtained from TG thermograms. ^2^ obtained from DTG thermograms.

**Table 3:** Water contact angles of pure PLLA and PLLA/TPC films.

PLLA/TPC (%*w*/*w*)	Water Contact Angle (°)
100/0	98.29 ± 4.57
99/1	95.41 ± 5.42
97.5/2.5	85.96 ± 4.74
95/5	78.49 ± 3.15
90/10	67.92 ± 4.36

## Data Availability

Data is contained within the article.
